# Diatom Mediated Production of Fluorescent Flower Shaped Silver-Silica Nanohybrid

**DOI:** 10.3390/ma14237284

**Published:** 2021-11-28

**Authors:** Piya Roychoudhury, Aleksandra Golubeva, Przemysław Dąbek, Michał Gloc, Renata Dobrucka, Krzysztof Kurzydłowski, Andrzej Witkowski

**Affiliations:** 1 Institute of Marine and Environmental Sciences, University of Szczecin, Mickiewicza 16a, 70-383 Szczecin, Poland; alexandra.golubeva@phd.usz.edu.pl (A.G.); przemyslaw.dabek@usz.edu.pl (P.D.); andrzej.witkowski@usz.edu.pl (A.W.); 2 Faculty of Materials Science and Engineering, Warsaw University of Technology, Wołoska 141, 02-507 Warsaw, Poland; michalgloc@wp.pl (M.G.); or renata.dobrucka@ue.poznan.pl (R.D.); 3 Department of Industrial Products and Packaging Quality, Institute of Quality Science, Poznań University of Economics and Business, al. Niepodległości 10, 61-875 Poznań, Poland; 4 Faculty of Mechanical Engineering, Białystok University of Technology, Wiejska 45c, 15-351 Białystok, Poland; k.kurzydlowski@pb.edu.pl

**Keywords:** diatom, silver-silica, nanohybrid, nanoflower, fluorescent particles

## Abstract

Fabrication of flower-like nanostructures are gaining attention because of their high surface/volume ratio and extensive adsorption capacity. In the present investigation, flower-shaped, autofluorescent silver-silica (Ag-SiO_2_) hybrid nanoparticles have been fabricated exploiting diatoms as a source of nanosilica. Two different species of *Gedaniella* including *G. flavovirens* and *G. mutabilis* showed their efficacy in synthesizing fluorescent Ag-SiO_2_ nanoflowers (NFs) and nanospheres (NSs) against 9 mM silver nitrate solution, respectively. The biogenic nanoconjugate (Ag-SiO_2_) was characterized by Uv-vis spectroscopy, energy dispersive X-ray spectroscopy (EDS), scanning (SEM) and transmission (TEM) electron microscopy. Production of Ag-SiO_2_ hybrid nanoparticle was confirmed by observing both Ag and Si signals from a single nanoparticle in an EDS study. The broad and single absorption band at ~420 nm in Uv-vis spectroscopy confirmed proper miscibility and production of hybrid nanoparticles. The Ag-SiO_2_ nanohybrids revealed autofluorescent property under the blue light region (excitation ~450–490 nm). SEM images of particles synthesized by *G. flavovirens* revealed the production of microscopic flower shaped Ag-SiO_2_ particles with several layers of petals. A TEM study confirmed that the synthesized Ag-SiO_2_ NFs are variable in size with 100–500 nm in diameter. Decolorization of methylene blue after exposure to Ag-SiO_2_ particles confirmed catalytic activity of synthesized nanostructures. This eco-friendly method provides a new dimension in nanobiotechnology for biogenesis of such hierarchical nanostructure in a cost-effective way.

## 1. Introduction

Silver nanoparticles (AgNPs) are well known for their extensive antimicrobial activity [1] and applicability in different physical fields as catalysts [2], biosensors [3], conductive adhesives [4], in water treatment [5], ink-jet printing [6], protein sensing [7] and solar cell optimization [8], etc. However, the Ag particles are less stable and tend to aggregate. To conquer this problem, AgNPs are needed to combine with some other nanoparticles. The mixture of one or more different nanoparticles with AgNPs which are structured to overcome the restrictions of mono metallic (Ag) nanoparticles are called hybrid Ag nanomaterials. The binding of AgNPs with some inert materials like silica makes the particles more effective and stable [9], helps to improve properties and to attain various functionalities. Recently, Ag-decorated silica nanoparticles have been synthesized by some chemical methods such as the wet-impregnation method [10] and metal-assisted chemical etching [11], which involves harmful chemicals like cetyltrimethylammonium bromide (CTAB) and hydrofluoric acid (HF). None of these techniques are environmentally friendly due to the involvement of hazardous chemicals. However, biogenic production of Ag-SiO_2_ NPs is required to avoid the involvement of toxic chemicals. Therefore, diatoms can be used as bioreagents for the biosynthesis of Ag-SiO_2_ NPs nanoparticles because diatom frustules contain nearly 88–90% of silica [12]. The biosilica, obtained from *Thalassiosira weissflogii*, *Pinnularia* sp. has already been modified with silver [13], and germanium [14] by chemical modification and metabolic insertion, respectively, to design hybrid multifunctional heterostructures. It has been reported that the diatoms, *Chaetoceros* sp., *Skeletonema* sp., *Thalassiosira* sp. [15], and *Navicula* sp. [16] are efficient in synthesizing AgNPs. Still, only a single report regarding the synthesis of hybrid Ag-SiO_2_ nanodendrites by *Halamphora subturgida* is available [17].

Among the different shapes, flower shaped nanoparticles are gaining attention because of their high adsorption capacity [18]. Nanoparticles which look like plant flowers under an electron microscope and having size ranges from 100–500 nm are known as nanoflowers (NFs), a newly developed structural category of various nanoforms [19,20,21]. The arrangement of several nanopetals on an axis creates this beautiful metallic floral morphology. The high surface to volume ratio of these complex nanostructures enhances the efficiency of the surface reaction [22]. The larger surface area of a small structure makes it suitable for multiple applications such as catalysts, biosensors, and drug delivery vehicles [23]. The flower-shaped nanoparticles have already been exploited in biosensor designing [24], drug delivery, photoimaging and diagnosis [25]. Microbial synthesis of flower shaped gold nanoparticles by *Bacillus subtilis* and *Bhargavaea indica* has been reported by Sreedharan et al., 2019 [26] and Singh et al., 2016 [25]. The higher plant mediated production of zinc oxide and copper oxide NFs was observed by Vinayagam et al., 2020 [27] and Siddiqi et al., 2019 [28], respectively. Green synthesis of silver NFs using starch as a reducing agent was performed by Ponsanti et al., 2020 [29]. No report is available regarding biogenic production of Ag-SiO_2_ NFs by diatoms.

It is common to use synthetic dyes as coloring agents in the textile, food, pharmaceutical and cosmetic industries. The non-biodegradable dyes are serious hazards for the environment. The dye from contaminated industrial wastes can enter the food chain and induce carcinogenicity. Currently, researchers are trying to exploit the high catalytic activity of various green synthesized nanoparticles in degradation of synthetic dyes. It has already been reported that silver nanoparticles are used with other reagents to enhance dye decolorlization [30]. The biosynthesized silver nanoparticles by the *Morinda tinctoria* leaf extract are capable of degrading 95% methylene blue within 72 h of reaction. Biogenic zinc oxide [31] and copper oxide [28] nanoflowers also showed high catalytic activity in degradation of methylene blue. Therefore, it can be said that biogenic Ag-SiO_2_ NFs will be an effective catalytic agent for methylene blue degradation.

In recent years, there has been an increase in research focused on the synthesis of nanoparticles which exhibit unique optical and electronic properties, especially with fluorescence emission. The fluorescent property of a nanoparticle depends on its size and composition [32]. Fluorescent nanoparticles are very useful in multimodal imaging of cancer detection [33]. For this reason, non-fluorescent nanoparticles are required to be labelled with some fluorescent dye like cyanine [34] or rhodamine [35]. The biological synthesis of auto-fluorescent silver and Cadmium sulfide nanoparticles by *Leea coccinea leaves* [36] and *Halobacillus* sp [37], respectively, has been reported. The fluorescent property of dendritic Ag-SiO_2_ particles synthesized by *Halamphora subturgida* has been described by Bose et al., 2021 [17]. Some authors reported that the luminescent properties of nanoparticles can be increased by coating them with silica beads [38]. Luminescent properties of the frustules of *Thalassiosira rotula*, *Cosinodiscus wailesii*, and *Cocconeis scutellum* have been demonstrated by Setaro et al., 2007 [39]. Therefore, it can be said that diatom could be an efficient bioreagent for production of auto-fluorescent metal-silica nanoconjugates.

In the present investigation, auto-fluorescent flower and spherical shaped Ag-SiO_2_ nanomaterials have been synthesized using diatom-strains isolated from the brackish waters of the Baltic Sea coasts, *G. flavovirens* and *G. mutabilis* with detail characterizations following the standard techniques along with their catalytic activities in degradation of methylene blue.

## 2. Materials and Methods

### 2.1. Chemicals

The chemicals used are silver nitrate (AgNO_3_ ≥ 99.0%, MW 169.86, Sigma-Aldrich, St. Louis, MO, USA); trisodium citrate dihydrate (HOC(COONa)(CH_2_COONa)_2_·2H_2_O ≥ 99.0%, MW 294.10, Sigma-Aldrich); hydrogen peroxide (H_2_O_2,_ 30%, Sigma-Aldrich) and methylene blue (C_16_H_18_CIN_3_S·XH_2_O, MW 319.85, Sigma-Aldrich).

### 2.2. Cultivation of Diatom

Two different species of *Gedaniella*—*G. flavovirens* (Takano) Ch.Li, Witkowski & Ashworth (SZCZCh1268) [40] and *G. mutabilis* (Grunow) Ch.Li & Witkowski (SZCZCh153) [40] were obtained from the Diatom Culture Collection, University of Szczecin, Poland. The chain diatoms were cultured at a salinity of 7‰ in the Guillard’s f/2 culture medium [41]. The strains were cultivated for 24 days at 20 °C under a 12:12 light: dark cycle, illuminated with 100 μmol photons m^−2^ s^−1^ of white light to determine the growth kinetics. The growth rate of *G. flavovirens* and *G. mutabilis* was checked by measuring dry weight of biomass (g L^−1^) and counting the number of cells (cells mL^−1^) on every two days up to 24th day of cultivation. The cells (cells mL^−1^) of each strain were counted under inverted NIKON eclipse TS100 microscope (Tokyo, Japan) at 400× magnification with Malassez counting chamber. Density of cells per mL was calculated following the formula of Edler and Elbrächter, 2010 [42],

Number of cells mL^−1^ = C × At/Af × F × V, (1)

[C = number of cells counted, At = total area of settling chamber (mm^2^), Af = area of a field (mm^2^), F = number of fields counted, V = volume of sample settled (mL)].

The biomass of *G. flavovirens* and *G. mutabilis* was harvested by centrifugation at 3000 rpm for 15 min and dried at 50 °C for two days. Dry weight of biomass (g L^−1^) was recorded on every other day from 1st to 24th day of cultivation. The regression curves of each strain were prepared and the specific growth rate ‘µ’ was calculated following the formula by Garci et al., 2000 [43],

(2) $${µ}_{i}=\frac{1}{t_{i+1}-t_{i}}\cdot \left[\ln\left(\frac{C_{i+1}}{C_{0}}\right)-\ln\left(\frac{C_{i}}{C_{0}}\right)\right]$$

where C is the biomass concentration at any time (t) and C_0_ is the initial biomass concentration.

### 2.3. Biogenesis of Ag-SiO_2_ Nanohybrid

The healthy cells of *G. flavovirens* and *G. mutabilis* from exponential growth phase were centrifuged at 5000 rpm for 5 min, later rinsed with double distilled water (ddH_2_O) to remove the excess of salt and recollected by centrifugation respectively. The thoroughly washed (200 mg) biomass of *G. flavovirens* and *G. mutabilis* was separately exposed to 50 mL of 9 mM AgNO_3_ solution with pH 4. The experimental setup was kept in dark at room temperature for 10 days. After 10 days of reaction, the resultant brown colored biomass was collected by centrifugation at 10,000 rpm for 15 min. The harvested biomass was rinsed with ddH_2_O water.

### 2.4. Documentation of Morphological Changes in Ag Treated Diatoms

Associated morphological changes in *G. flavovirens* and *G. mutabilis* after 10 days of reaction with AgNO_3_ were documented by capturing light microscopic images with ZEISS Axioscope.A1 (Jena, Germany) at 400× magnification. To understand the surface ornamentation of control and Ag treated *G. flavovirens* and *G. mutabilis*, the SEM images have been taken using a Hitachi SU8020 (Hitachi, Tokyo, Japan). For SEM study, the samples were dried overnight at room temperature and coated with a 5 nm thick chromium layer.

### 2.5. Extraction of Nanomaterials

The extraction of Ag-SiO_2_ nanohybrid was performed following the protocol of Bose et al., 2021 [17]. The nanoparticle loaded biomass of *G. flavovirens* and *G. mutabilis* was suspended in 7.5 mM sodium citrate solution for 24 h. Then the suspensions were vortexed for 30 min and filtered. The filtered suspensions were used for further characterizations.

The control cells of experimental taxa were also treated with sodium citrate to prepare the sodium citrate extracts of control biomass. The healthy biomass (5 mg) of *G. flavovirens* and *G. mutabilis* was sonicated with 1 mL of 7.5 mM sodium citrate solution for 25 min by a Hielscher UP100H ultrasonic processor (Teltow, Germany). Furthermore, the solution was centrifuged at 10,000 rpm for 10 min, and the supernatant was collected and stored at −20 °C for further utilization.

Likewise, the 30% of H_2_O_2_ treated, cleaned frustules (5 mg) of *G. flavovirens* and *G. mutabilis* were also sonicated with 7.5 mM sodium citrate solution for 25 min followed by a centrifugation at 3000 rpm for 5 min to extract nanosilica from the frustules of experimental strains.

### 2.6. Uv-Vis Spectroscopy

The sodium citrate extracts of Ag-SiO_2_ NPs loaded *G. flavovirens* and *G. mutabilis* were subjected to Hach DR6000 Benchtop UV-visible spectrophotometer (Hach, Loveland, CO, USA) for optical measurements. To determine the maximum absorbance of biosynthesized Ag-SiO_2_ NPs, the extracted nano-suspensions were analyzed with a Uv-vis spectrophotometer in the wavelength range of 300–1100 nm. Similarly, the Uv-vis spectra of sodium citrate extracts of control biomass and H_2_O_2_ treated, cleaned frustules of *G. flavovirens* and *G. mutabilis* were also recorded in the same wavelength range. A comparative spectral analysis among sodium citrate extracts of control, Ag-SiO_2_ NPs loaded and H_2_O_2_ treated frustules were performed.

### 2.7. Elemental Composition and Electron Microscopic Analysis of Synthesized Particles

Elemental compositions of extracted brown suspensions from *G. flavovirens* and *G. mutabilis* were measured using the EDS technique. A drop of suspension was dried on a carbon-coated copper grid and the grid was analyzed with Hitachi STEM S5500 attached with EDS detector. The arrangement of silica and silver in the nanostructures was achieved by EDS mapping, which was performed using NSS Thermo Scientific software. Measurements were performed with an accelerating energy 30.0 kV. From the EDS spectra, atom % of silicon, oxygen, and silver have been determined. Using the same grid and same instruments, SEM and TEM images have been captured to understand the shape and size of the synthesized particles.

### 2.8. Fluorescent Microscopy

The changes in fluorescent properties of control and Ag-SiO_2_ NPs loaded cells of *G. flavovirens* and *G. mutabilis* were observed by an Axioscope A1 Zeiss fluorescence microscope (excitation: 450–490 nm and emission: 515 nm). The autofluorescence properties of synthesized Ag-SiO_2_ NPs were checked by taking a drop of extracted brown suspension on a glass slide with Zeiss imager. Z2 with an excitation at 450–490 nm and emission at 515 nm spectra.

### 2.9. Catalytic Activity of Synthesized Nanostructures

The catalytic activity of synthesized flower and spherical shaped Ag-SiO_2_ NPs by *G. flavovirens* and *G. mutabilis* respectively was investigated by recording dye degradation (methylene blue) with time. The 1000 mL stock solution of 10 ppm methylene blue was prepared. The extracted suspensions of Ag-SiO_2_ NPs from diatoms were lyophilized, and then the concentration of the synthesized particles was calculated as milligram per milliliter. To determine the dye degradation kinetics, 2 mg of flower and spherical shaped Ag-SiO_2_ NPs were separately added to 10 ppm, 20 mL of methylene blue solution and stirred for 30 min at room temperature. The dye decolorlization was determined at 0, 15, 30, 45, 60, 120 min time intervals with a Uv-vis spectrophotometer in the wavelength range between 500–900 nm. A control dye sample was also monitored without the addition of nanoparticles. The entire experiment was performed under light. The amount of dye degradation was calculated using the absorbance value at 660 nm. The percentage of methylene blue degradation was determined by the following formula [44]:

% of dye degradation = 100 × (C_0_ − C)/C_0_

where C_0_ is the initial and C is the concentration of methylene blue solution after specific time of reaction.

## 3. Results

### 3.1. Culturing of Diatoms and Growth Determination

**Figure 1:**
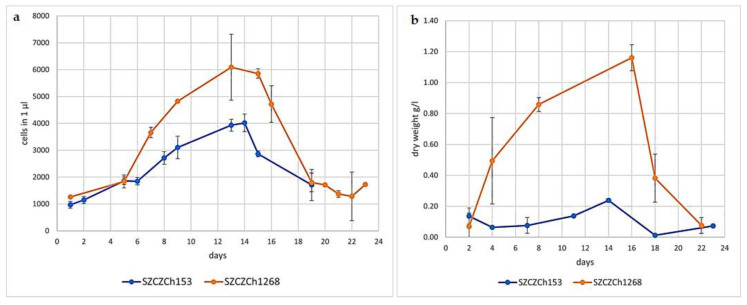
Growth curves for *G. flavovirens* (SZCZCh1268) and *G. mutabilis* (SZCZCh153): (**a**) density of cells in 1 mL per day; (**b**) dry biomass (g·L^−1^).

*G. flavovirens* and *G. mutabilis* showed distinct lag, exponential, stationary and death phases during batch culture in Guillard medium (Figure 1). It was observed that the growth of *G. flavovirens* has started after the second day of inoculation, followed by the exponential phase until the eighth day of cultivation. The maximum specific growth rate on the exponential phase was calculated as 0.38 d^−1^ (per day). A stationary phase was observed from the eighth to 16th days with a specific growth rate µ_stat_ = 0.04 d^−1 ^ and on the 16th day, the death phase started (Figure 1a,b). During cultivation of *G. mutabilis*, the lag phase of *G. mutabilis* was recorded until the fourth day and the exponential phase was observed up to the 11th day with maximum specific growth µ_max_ = 0.16 d^−1^. From the 12th day, the stationary phase started (specific growth rate µ_stat_ = 0.01 d^−1^), and after 15 days of cultivation, the death phase was initiated (Figure 1a,b). Therefore, it can be said that the maximum biomass yield for *G. flavovirens* (1.16 ± 0.084 gL^−1^) and *G. mutabilis* (0.24 ± 0.011 gL^−1^) was obtained on 16th and 14th day of cultivation respectively.

### 3.2. Associated Morphological Changes in Ag^+^ Exposed Gedaniella

**Figure 2:**
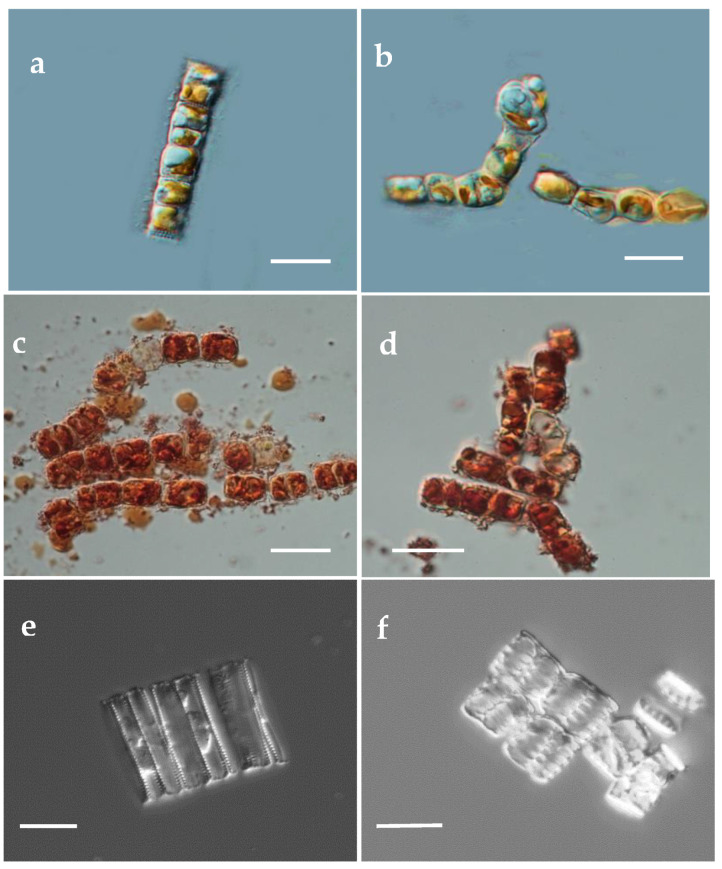
Light microscopic images of control (**a**,**b**), Ag treated (**c**,**d**) and cleaned frustules (**e**,**f**) of *G. flavovirens* and *G. mutabilis* respectively [Scale bar 5 µm].

**Figure 3:**
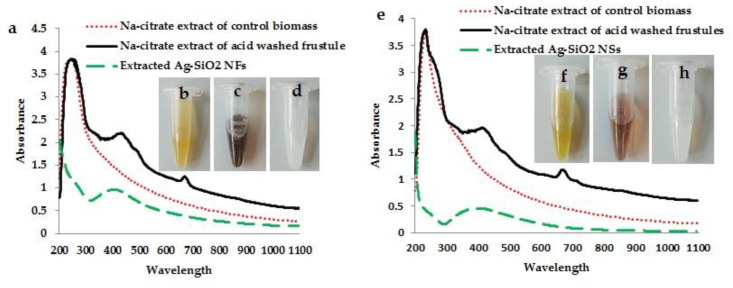
Absorption bands of control, Ag treated, and H_2_O_2_ washed frustules extracts of *G. flavovirens* in Uv-vis spectroscopy (**a**). Uv-vis spectra of sodium citrate extracts of control, Ag treated, and H_2_O_2_ washed frustules of *G. mutabilis* in 200–1100 nm wavelength range (**e**). Sodium citrate extracts of control cells (**b**,**f**), metal treated cells (**c**,**g**) and cleaned frustules (**d**,**h**) of *G. flavovirens* and *G. mutabilis*, respectively.

The morphological changes in *G. flavovirens* and *G. mutabilis* due to Ag^+^ stress was preliminarily confirmed by observing color change in biomass. The yellowish-green colored cells of *G. flavovirens* (Figure 2a) and *G. mutabilis* (Figure 2b) turned dark golden brown in color after 10 and 4 days of reaction with 9 mM Ag^+^ solution (Figure 2c,d), respectively. The color change was initiated in the biomass of *G. flavovirens* and *G. mutabilis* after four and one days of exposure in 9 mM aqueous AgNO_3_ solution, respectively. No further color change in metal treated *G. flavovirens* and *G. mutabilis* was observed after 10 and 4 days of reaction, respectively. It was documented that under Ag^+^ stress, both strains lost all their pigment content (chlorophyll and carotenoids), which was further confirmed by Uv-vis spectroscopy (Figure 3a,e). The loss of cellular integrity in *G. flavovirens* and *G. mutabilis* signified silver toxicity.

**Figure 4:**
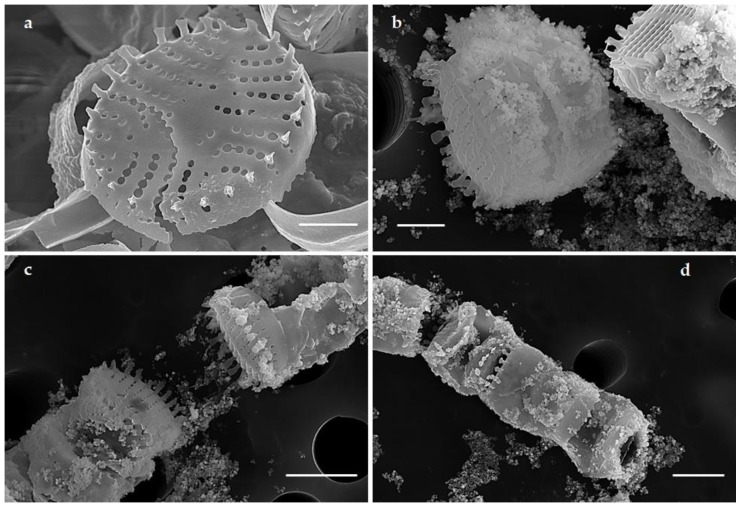
SEM images showing surface morphology of control (**a**) and silver treated (**b**–**d**) frustules of *G. flavovirens* and confirmed the extracellular deposition of nanoparticles in aggregated forms [scale bar 1 µm].

**Figure 5:**
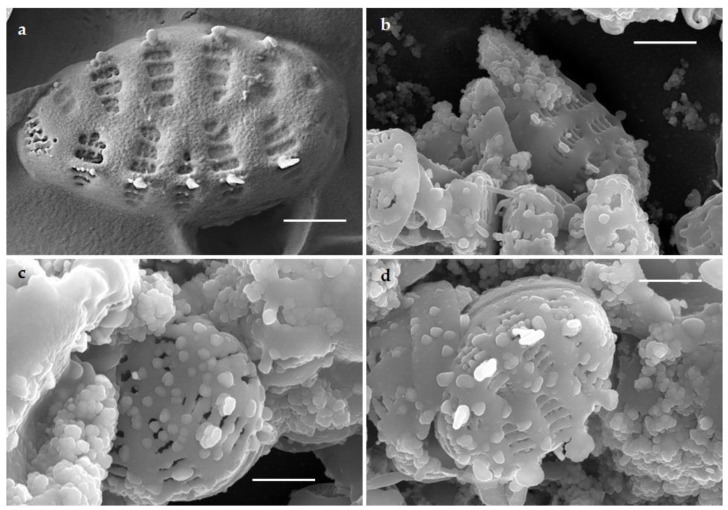
SEM images of control (**a**) and silver treated (**b**–**d**) frustules of *G. mutabilis* and confirmed the deposition of nanoparticles on surfaces of Ag treated cells [scale bar 1 µm].

SEM images revealed the surface topography of control and AgNO_3_ treated cells of *G. flavovirens* and *G. mutabilis*. SEM micrographs illustrated the production of nanostructures and their deposition on siliceous frustules of *G. flavovirens* and *G. mutabilis*. Surfaces of Ag-treated frustules were completely covered with nanoparticles, which differs from the surface ornamentation of control cells of *G. flavovirens* (Figure 4) and *G. mutabilis* (Figure 5).

### 3.3. Optical Measurements

In Uv-vis spectroscopy, sodium citrate extracts of control biomasses of both *G. flavovirens* and *G. mutabilis* showed three distinct peaks at 270, 450, and 663 nm (Figure 3a,e). These three consecutive peaks at 270, 450, 663 nm revealed the presence of silica, carotenoids and chlorophyll, respectively, in untreated cells. The sodium citrate extracts of H_2_O_2_ washed frustules of *G. flavovirens* and *G. mutabilis* showed maximum absorbance at 270 nm and confirmed the presence of only silica particles without any pigment trace (Figure 3a,e). The brown suspensions extracted from Ag-treated *G. flavovirens* and *G. mutabilis* showed a characteristic peak at about ~420 and 415 nm, respectively. These broad and single absorption bands confirmed the presence of Ag-SiO_2_ hybrid nanoparticles and the absence of free silica particles and any other pigment content in extracted brown suspensions (Figure 3a,e).

### 3.4. Electron Microscopic Study

**Figure 6:**
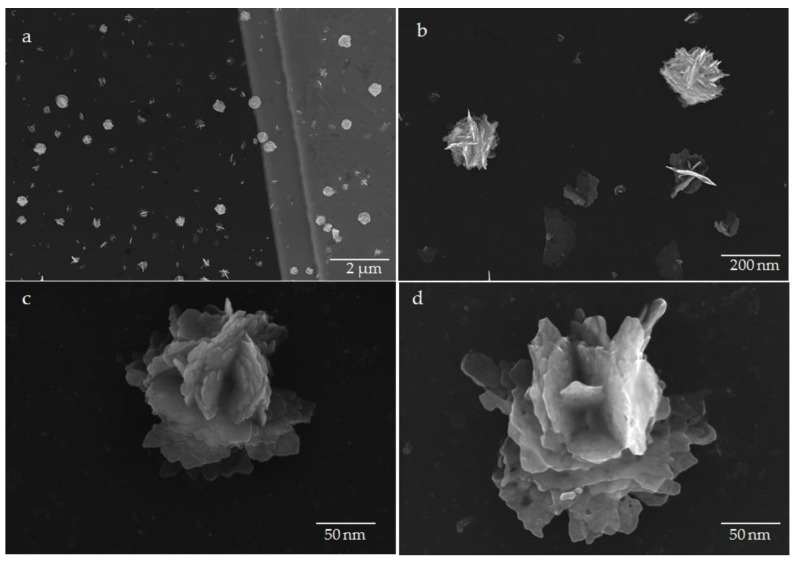
SEM images of flower shaped Ag-SiO_2_ nanohybrid synthesized by *G. flavovirens* in variable magnifications (**a**–**d**).

**Figure 7:**
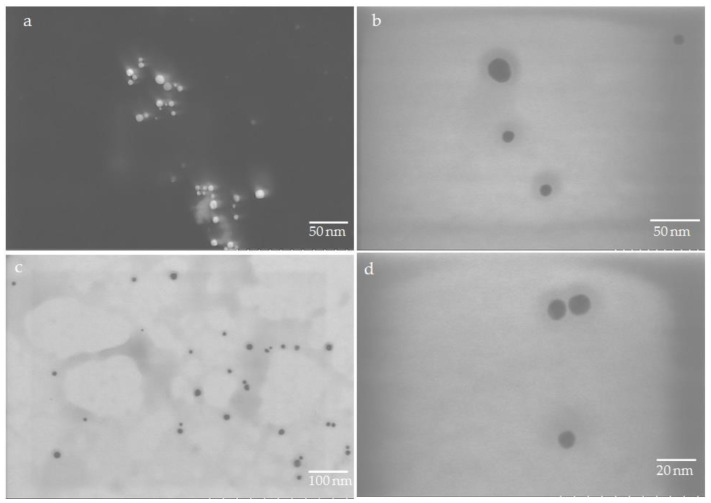
SEM (**a**) and TEM (**b**–**d**) micrographs of spherical shaped Ag-SiO_2_ nanohybrid synthesized by *G. mutabilis.*

The SEM images of biogenic particles by *G. flavovirens* confirmed the production of predominant flower-like structures with several layers of petals (Figure 6a–d). The petals are associated with each other, attached in the center and developed a unique flowerlike morphology. The single petal length was approximately 150–200 nm and the diameter of the petals were different from the roots to the tips. The SEM micrographs confirms that the particles synthesized by *G. mutabilis* are all spherical in nature with different sizes (Figure 7a).

**Figure 8:**
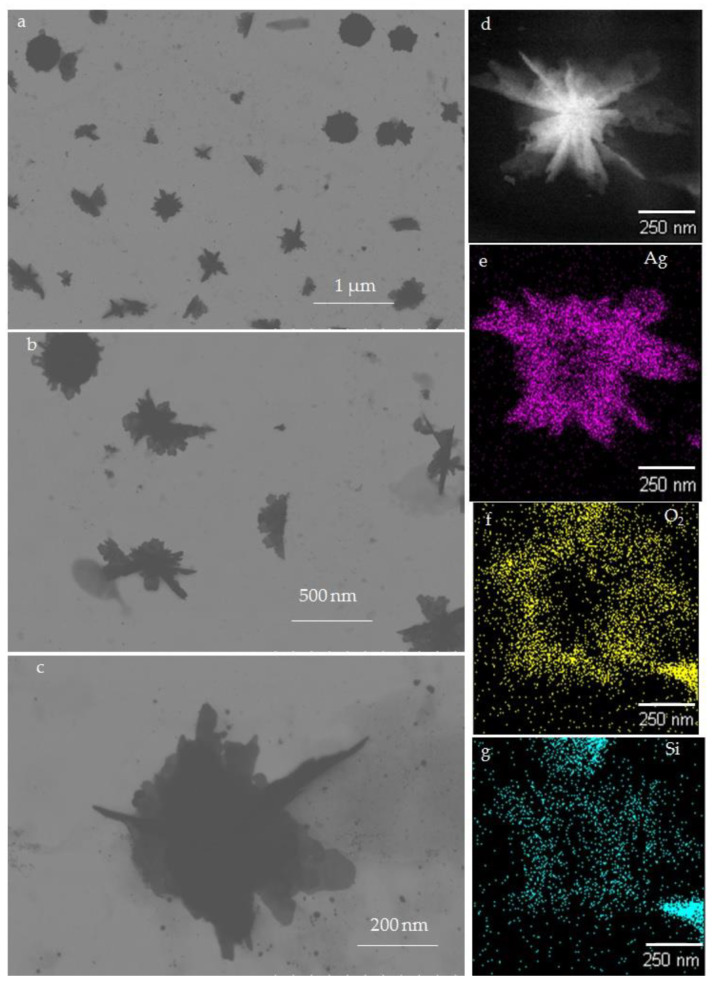
TEM images of flower shaped Ag-SiO_2_ nanohybrid synthesized by *G. flavovirens* in different magnifications (**a**–**c**). EDS mapping (**d**–**g**) showing the distribution pattern of Ag, Si and O_2_ in all over the Ag-SiO_2_NF.

TEM images revealed the size range of biofabricated Ag-SiO_2_ NFs by *G. flavovirens* as between ~100 to 500 nm (Figure 8a–c). TEM micrographs also confirmed that each petal has a sharpened tip (Figure 8). It was documented by a TEM study that the biosynthesized Ag-SiO_2_ particles by *G. mutabilis* are all spherical in nature with a 10–40 nm diameter range (Figure 7b–d).

### 3.5. Elemental Composition

**Figure 9:**
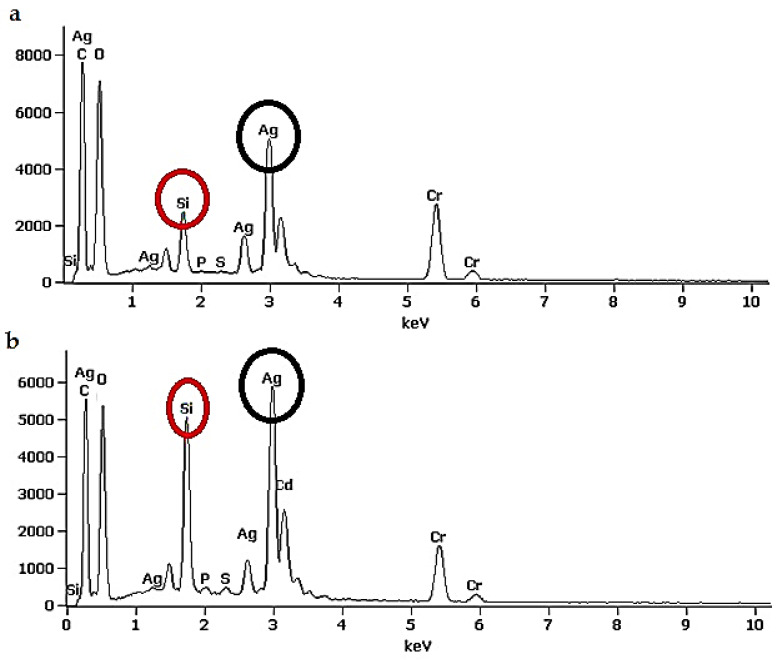
EDS spectra of synthesized nanohybrid (Ag-SiO_2_) by *G. flavovirens* (**a**) and *G. mutabilis* (**b**). Spectra (**a**,**b**) showing presence of Ag and Si both signals in a single point of synthesized particles and confirming production of Ag-SiO_2_ nanohybrid.

The EDS spectra of suspensions extracted from Ag-treated *G. flavovirens* and *G. mutabilis* are shown in Figure 9. The spectra of nanostructures synthesized by *G. flavovirens* and *G. mutabilis* both showed three distinct peaks, which corresponds to the signals of silicon, oxygen, and silver from a single point, respectively. On the other hand, EDS spectra of cleaned frustules extracts of *G. flavovirens* and *G. mutabilis* showed the signals of silicon and oxygen only. The spectra revealed an approximate atom percentage of synthesized particles by *G. flavovirens* (2.3% silicon, 61.3% oxygen, and 4.4% silver) and *G. mutabilis* (5% silicon, 54.3% oxygen, and 5% silver). EDS analysis revealed that the particles synthesized by *G. flavovirens* and *G. mutabilis* against 9 mM AgNO_3_ solution were hybrid in nature.

Elemental mapping of a single Ag-SiO_2_ NF (Figure 8d–g) illustrated the distributional pattern of the elements (Silicon, oxygen and silver) all over the synthesized nanostructure. It was confirmed from EDS mapping (Figure 8e) that Ag particles are distributed all around the flower-shaped particle with a higher concentration at the center. Silicon and oxygen were especially dispersed in the peripheral region of Ag-SiO_2_ NF (Figure 8f–g).

### 3.6. Fluorescent Microscopic Analysis

**Figure 10:**
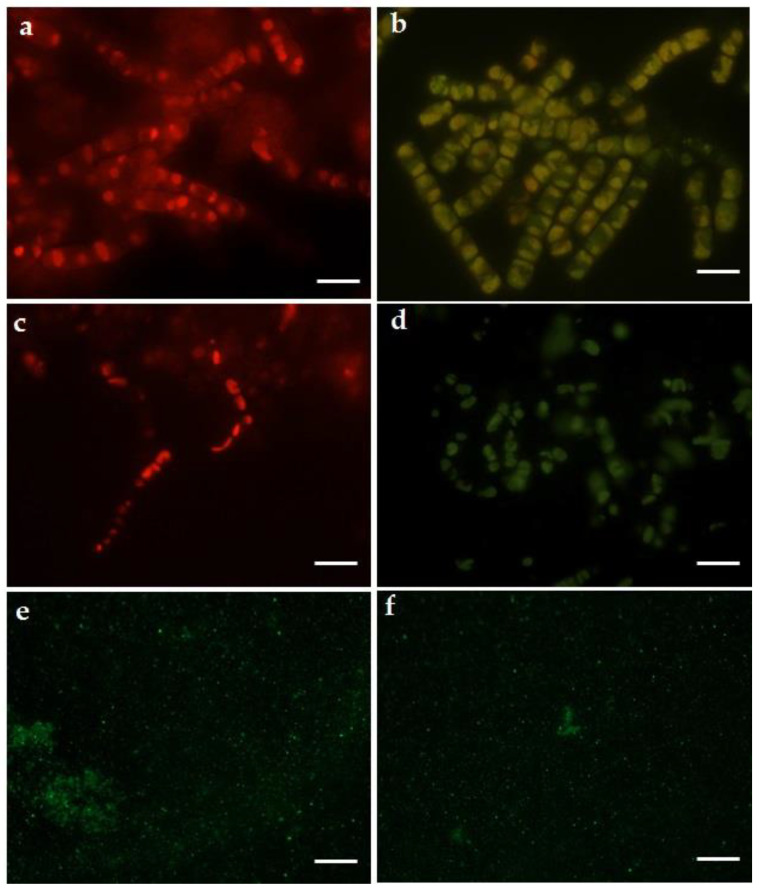
Fluorescent images of control (**a**,**c**) and Ag treated (**b**,**d**) *G*. *flavovirens* and *G*. *mutabilis* respectively. Fluorescent images of Ag-SiO_2_ nanohybrid synthesized by *G*. *flavovirens* (**e**) and *G*. *mutabilis* (**f**) [scale bar 1 µm].

Under a fluorescent microscope, in the blue light region (450–490 nm), control cells of *G. flavovirens* and *G. mutabilis* showed red fluorescent property due to presence of chlorophyll (Figure 10a,b). However, after two days of reaction with AgNO_3_ solution, a few cells of *G. flavovirens* and *G. mutabilis* started to fluoresce green in contrast to the red fluorescent property of other cells. After 10 days of Ag^+^ exposure, it was observed that all cells of *G. flavovirens* and *G. mutabilis* showed only green fluorescence with a greater intensity (Figure 10c,d). The extracted nanoflowers from *G. flavovirens* and the nanosphere from *G. mutabilis* also showed autofluorescence property with excitation spectra at 450–480 nm and emission spectrum at 530 nm (Figure 10e,f).

### 3.7. Catalytic Activity of Flower and Spherical Shaped Ag-SiO_2_ Nanohybrid

**Figure 11:**
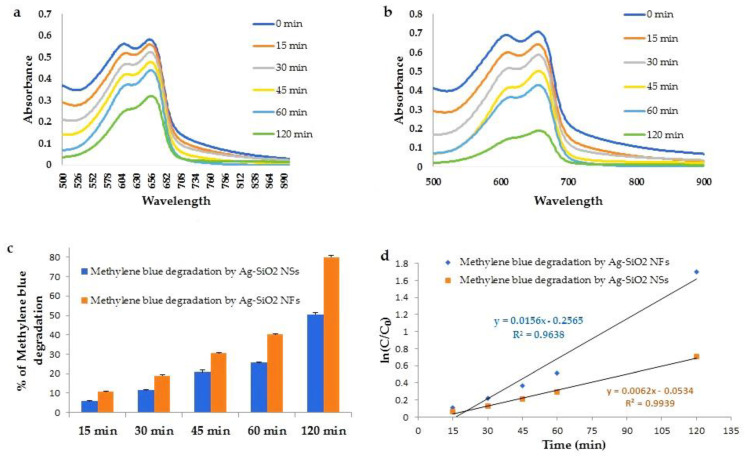
Absorbance spectra of methylene blue with time after exposure to Ag-SiO_2_ NSs (**a**) and Ag-SiO_2_ NFs (**b**). Degradation percentage of methylene blue after treatment with Ag-SiO_2_ particles (**c**). First-order kinetic plot of ln(C_0_/C) versus time for the degradation of methylene blue (**d**).

Biogenic flower- and spherical-shaped Ag-SiO_2_ particles synthesized by *G. flavovirens* and *G. mutabilis*, respectively, both showed positive responses in degradation of methylene blue. Methylene blue degradation after exposure to Ag-SiO_2_ particles was primarily confirmed by a color change of the blue solution. Initially, the color of the dye was deep blue and turned greenish after 15 min of reaction and gradually faded with time. Spectroscopic analysis showed a sharp decrease of peak for methylene blue with an increase of exposure time, which indicated degradation of methylene blue (Figure 11). After 120 min of reaction with flower shaped Ag-SiO_2_ particles, 80% dye degradation and with spherical Ag-SiO_2_ particles, 50% dye degradation were observed (Figure 11). The linear plot for ln(C_0_/C) against time follows the kinetic theory and rate constants of methylene blue degradation by Ag-SiO_2_ NFs and Ag-SiO_2_ NSs are 0.256 and 0.053 min^−1^, respectively.

## 4. Discussion

Biosilica formation is a characteristic feature of all diatoms which forms the outer covering of cells and are referred to as frustules. The porous structural morphology of frustules generates a unique, species-specific ultrastructural pattern. The diatom frustule itself plays the role of reducing agent in metal ion reduction and consequent production of metal nanoparticles [17]. Association of biogenic nanoparticles with the surfaces of frustules has already been documented in *Navicula* [16] and *Halamphora* [45]. Here also, synthesized nanostructures deposited on the exterior side of frustules of *G. flavovirens* and *G. mutabilis* was confirmed by SEM study. It was evident that the color of nanosilver varies from light yellow to dark brown in color [46]. In this study, the color changes in diatom cells from yellowish brown to dark golden brown after AgNO_3_ exposure indicated reduction of Ag^+^ and the subsequent production of silver nanoparticles. It was clear that the synthesis of nanomaterials by *G. flavovirens* and *G. mutabilis* cells started after four and one days of reaction and was completed after 10 and 4 days of reaction, respectively, without showing any further color change. However, the color change couldn’t determine the formation of hybrid Ag-SiO_2_ nanoparticles. The synthesis of SiO_2_ conjugated hybrid Ag nanoparticles was later confirmed by Uv–vis spectroscopy, EDS spectra and mapping.

An EDS study analytically identifies the elements (elemental composition) present in any given material. Here, the characterization of brown suspensions extracted from Ag^+^ treated *Gedaniella* by EDS determined the presence of three different elements (Ag, Si, O) in a single particle and confirmed that the synthesized particles (Ag-SiO_2_) were hybrid in nature. On the other hand, production of hybrid nanomaterials was further confirmed by observing a single plasmon band (~410 nm) in Uv-vis spectroscopy. It is well known that pure silica and silver particles show an intense peak in Uv-Vis spectroscopy at ~230–270 nm [47] and ~400–450 nm, [48] respectively. The single and broad absorption band in the visible region confirms the absence of free SiO_2_ particles and their association with Ag particles. If pure silver and silica nanoparticles are mixed physically, two plasmon bands are expected to be observed. Therefore, the EDAX and Uv-vis spectroscopy confirmed the conjugation between two different nanoforms (Ag and SiO_2_) and the production of a nanohybrid (Ag-SiO_2_). Here, particles extracted from cleaned frustules showed a distinct peak at 230 nm, which revealed that frustules are the source of silica particles.

Ag particles are required to be immobilized with silica to increase their stability. However, the chemical process to synthesize SiO_2_ associated Ag particles does not follow the ecofriendly route. Here, diatoms-*G. flavovirens* and *G. mutabilis* have been used as a source of silica for production of biocompatible Ag-SiO_2_ particles in a cost-effective way without involving any hazardous chemicals. Both strains, *G. flavovirens* and *G. mutabilis*, showed efficiency in reduction of Ag^+^ ions due to the presence of different reducing molecules inside the diatom cells like proteins [49], carotenoids [50], polysaccharides [51], etc. A few authors have reported that frustules can directly act as a reducing agent due to the presence of the silanol group. Oxidation of silanol helps in the reduction of metal ions and results in the consequent production of metal nanoparticles [17]. It is also reported that the amide group, present on the frustule surface, takes part in the metal ion reduction process [51]. Frustules are also a source of glycoprotein and frustulin [52]. Therefore, it can be said that all these reducing agents might be involved in the reduction of Ag^+^ ions. Besides that, in a silica deposition vesicle, continuous amorphous nanosilica is formed from monosilicic [Si(OH)_4_] and disilicic [Si_2_O(OH)_6_] acids. It has also been reported that silaffins proteins play a major role in the conversion of nanosilica from silicic acid [53]. Eventually, the simultaneous production of silica and silver nanoparticles resulted in the association of Ag and SiO_2_ particles and the subsequent production of the spherical- and flower-shaped Ag-SiO_2_ nanohybrid.

In this study, it was observed that two different species of a single genus viz. *G. flavovirens* and *G. mutabilis* synthesized flower and spherical shaped Ag-SiO_2_ nanoconjugates, respectively, in the same experimental conditions. SEM and TEM studies revealed that the spherical shaped particles were synthesized by the whole biomass of *G. mutabilis,* whereas the Ag-SiO_2_ nanohybrid synthesized by *G. flavovirens* are all flower-shaped in nature. The fabrication of various shaped nanoparticles by different species of the same genus is common in green synthesis. *Sargassum cinereum* [54] is efficient in synthesizing triangular and spherical silver nanoparticles, whereas *Sargassum longifolium* [55] synthesized cubical-shaped silver nanoparticles in the same reaction condition. The synthesis of spherical and rod-shaped gold nanoparticles by *Phormidium valderianum* and the production of spherical- to irregular-shaped gold nanoparticles by *Phormidium tenue* has been reported [56]. The formation of distinct shapes depends on precursor type and reaction conditions and time. Here, the same reaction conditions were maintained for both strains, but the reduction time varied for the two species. *G. mutabilis* took four days to complete the reaction procedure, whereas *G. flavovirens* turned dark brown in color after 10 days of reaction. Naturally, the biosynthesis of nanoparticles is a stress response of bio-organisms against toxic substances. In response to the toxic substances, stress enzymes are released (reducing agents) in diatom cells which interact with the metal ions through electrostatic interactions. The stress tolerance and response vary between species.

The auto-fluorescence properties of the resultant spherical- and flower-shaped Ag-SiO_2_ nanohybrid confirmed the association of Ag particles with SiO_2_. SiO_2_ always shows green fluorescence at the 450–490 nm excitation wavelength with an emission spectrum at 515 nm [17]. Here, synthesized Ag-SiO_2_ also showed fluorescent properties in the same excitation and emission wavelength range. Likewise, the Ag-treated cells of *G. flavovirens* and *G. mutabilis* showed only a green fluorescent property under the blue light region due to the presence of silica [17]. The disappearance of red fluorescence in particle-loaded cells of *G. flavovirens* and *G. mutabilis* confirmed damage of chlorophyll due to AgNO_3_ stress. The unique optical properties of nanoparticles can be achieved in biogenesis by using diatom frustules which are composed of nearly 88–90% of silica as a bioreagent. The luminescent Ag-SiO_2_ nanostructures can be utilized in bioimaging in the future.

Nanoparticles are good catalysts because of their high surface to volume ratio and specific surface plasmon resonance [57]. Any plasmonic excitation helps them to release surface electrons from the outermost band [58]. It can be said that methylene blue is reduced to a colorless compound by accepting electrons from Ag-SiO_2_ nanoparticles. The Ag-SiO_2_ NFs showed more efficiency in methylene blue decolorization than Ag-SiO_2_ NSs because of their larger surface area.

Due to their high surface reaction capability, flower-shaped nanoparticles have exciting potential applications in the field of nanobiotechnology compared to other nanostructures. These hierarchical structures have been developed by the continuous aggregation of Ag-SiO_2_ particles. The self-assembly of Ag-SiO_2_ nanohybrids resulted in nanopetals as basic building blocks of nanoflowers. With the prolongation of reaction time, the synthesized nanopetals became interconnected with each other and eventually their arrangement on an axis led to the formation of flower shaped nanoparticles. Usually, the synthesis of flower-shaped nanoparticles involves multi-step protocols, expensive chemicals, and reagents. In this study, a simple technique has been explained to synthesize Ag-SiO_2_ NFs in a cost-effective way.

## 5. Conclusions

In this study, an eco-friendly, cost-effective and simple technique has been described to synthesize diatom-assisted Ag-SiO_2_ conjugate nanoparticles. A comparative analysis between two different species (*G. flavovirens* and *G. mutabilis*) of a single genus as per their efficacy in synthesizing Ag-SiO_2_ nanohybrid has been completed. It was observed that Ag-SiO_2_ nanohybrid production against AgNO_3_ solution is possible by using *G. flavovirens* and *G. mutabilis* as reducing agents. It was noted that *G. mutabilis* is capable of synthesizing the spherical-shaped Ag-SiO_2_ nanohybrid within four days of reaction, whereas, *G. flavovirens* showed efficiency in synthesizing flower-shaped Ag-SiO_2_ nanohybrids after 10 days of Ag exposure in the same reaction conditions. Therefore, it can be hypothesized that metal tolerance mechanism and metal ion interaction patterns vary in every species. Furthermore, the production of distinct shaped nanoparticles is possible by using a specific reducing agent. The strain *G. flavovirens*, has been characterized as an efficient bioreagent for the production of biocompatible, flower-shaped Ag-SiO_2_ conjugate nanoparticles. The resultant structures can be exploited in various fields such as bio-sensing SERS detection and electronic device designing, as well as in medical fields. Synthesized Ag-SiO_2_ NFs can degrade 80% methylene blue within 120 min of reaction. This catalytic activity of flower-shaped Ag-SiO_2_ particles can be exploited in a synthetic dye removal technique from polluted water. The flower- and spherical-shaped Ag-SiO_2_ nanocomposites showed fluorescent properties which would be useful in bio-imaging. It can be concluded that diatoms are a potential source for synthesizing highly stable metal-silica hybrid nanoparticles with luminescent properties.

## Data Availability

Not applicable.
